# Parents' Knowledge, Risk Perception and Willingness to Allow Young Males to Receive Human Papillomavirus (HPV) Vaccines in Uganda

**DOI:** 10.1371/journal.pone.0106686

**Published:** 2014-09-09

**Authors:** Wilson Winstons Muhwezi, Cecily Banura, Andrew Kampikaho Turiho, Florence Mirembe

**Affiliations:** 1 Department of Psychiatry, School of Medicine, Makerere University College of Health Sciences, Kampala, Uganda; 2 Child Health and Development Center, School of Medicine, Makerere University College of Health Sciences, Kampala, Uganda; 3 Department of Obstetrics and Gynecology, School of Medicine, Makerere University College of Health Sciences, Kampala, Uganda; Federal University of São Paulo, Brazil

## Abstract

The Ministry of Health in Uganda in collaboration with the Program for Appropriate Technology for Health (PATH) supported by Bill and Melinda Gates Foundation in 2008–2009 vaccinated approximately 10,000 girls with the bivalent humanpapilloma virus (HPV) vaccine. We assessed parent's knowledge, risk perception and willingness to allow son(s) to receive HPV vaccines in future through a cross-sectional survey of secondary school boys aged 10–23 years in 4 districts. 377 questionnaires were distributed per district and 870 were used in analysis. Parents that had ever heard about cervical cancer and HPV vaccines; those who would allow daughter(s) to be given the vaccine and those who thought that HPV infection was associated with genital warts were more willing to allow son(s) to receive the HPV vaccine. Unwilling parents considered HPV vaccination of boys unimportant (p = 0.003), believed that only females should receive the vaccine (p = 0.006), thought their son(s) couldn't contract HPV (p = 0.010), didn't know about HPV sexual transmissibility (p = 0.002), knew that males could not acquire HPV (p = 0.000) and never believed that the HPV vaccines could protect against HPV (p = 0.000). Acceptance of HPV vaccination of daughters and likelihood of recommending HPV vaccines to son(s) of friends and relatives predicted parental willingness to allow sons to receive HPV vaccines. Probable HPV vaccination of boys is a viable complement to that of girls. Successfulness of HPV vaccination relies on parental acceptability and sustained sensitization about usefulness of HPV vaccines even for boys is vital.

## Introduction

Humanpapilloma Virus (HPV) infections are among the most common sexually transmitted infections (STIs) worldwide [3]. HPV infection is generally asymptomatic and in most cases, self-limiting. However, it can result in HPV type-specific clinical sequelae, including cervical neoplasia, genital warts; anogenital, head and neck cancers [8]. Efforts to prevent HPV infection have advanced over the years to a point of discovering appropriate vaccines [14], [17].

In Uganda, the Ministry of Health (MoH) in collaboration with PATH and supported by the Bill and Melinda Gates Foundation vaccinated approximately 10,000 girls in two districts with the bivalent HPV vaccine in 2008 and 2009 to demonstrate a viable strategy of giving the vaccine using existing public health delivery systems [33]. There are two vaccine types; the quadrivalent targeting HPV types 6, 11, 16 and 18 [14] and the bivalent targeting HPV types 16 and 18 [17]. Both can be given to females and males to prevent HPV infection [1], [2], [29]. Research shows that HPV vaccines should reduce the incidence of cervical cancer by 70%, genital warts by 90% and universal protection if administered to people not previously exposed to the implicated HPV types [10]. During preparation of the Ugandan population for HPV vaccination of girls, many stakeholders queried the exclusion of boys from vaccination against what was explained to be an STI.

HPV types 6, 11, 16, and 18 are known to be prevalent in both cervical and non-cervical diseases [26], [28], [30], [35]. Similar to women, men infected with these types experience a significant morbidity and potential mortality from HPV-related diseases. HPV types 6 and 11 are known to be strongly associated with genital warts, a common STI affecting both women and men [20]. Other than protection against HPV infection and anogenital warts, the quadrivalent vaccine is also known to target prevention of HPV 6 and 11, often associated with external genital lesions in young men, anal cancer, oral cancer and penile cancer [22], [29], [32]. Research has demonstrated that both the quadrivalent and bivalent HPV vaccines stimulate immunogenicity in males and females [2], [29].

Information regarding HPV infection and HPV-associated diseases is often directed at young women and parents of girls. Consequently, many men tend to be ill-informed about HPV and are unaware of its consequences for male health [10], [32]. In interpreting acceptability findings from male populations, it is important to contextualize the overall poor knowledge about HPV infection, disease, and transmission. Although females clearly benefit from receiving the HPV vaccine, males could also benefit from being vaccinated against low- and high-risk HPV types. Although correlates of HPV vaccine acceptance have been identified for mid-adult women [10], correlates of HPV vaccine acceptance among men in Uganda are yet to be described.

Like elsewhere, there are still many questions about HPV vaccines including; acceptability of vaccinating boys and girls, alternative delivery models of HPV vaccines, influence of HPV vaccination on sexual behaviors and the impact of sensitizing adolescents about HPV vaccination in Uganda. The study assessed parents' knowledge and perception of their son(s) risk to HPV infection and willingness to allow their son(s) to receive probable HPV vaccines in future as a complement to HPV vaccination of females.

## Study Methods

Ethical approval clearance to carry out the study was obtained from Makerere University School of Medicine Research and Ethics Committee and Uganda National Council for Science and Technology Committee on study of Human Subjects; District Directors of Health Services, District Education Officers and authorities in charge of immunization in the concerned districts. Data reported does not arise from a clinical study and is not from patients. Parents that participated in the study gave written informed consent. Parental refusal to participate was respected. The overall conduct of the research adhered to Helsinki Declaration [36]. Any study participant who demanded more information or needed other specialist attention was referred to an appropriate service provider.

### Study Design and Study Areas

In the study, a cross-sectional research design was used. The study was done in 4 districts, 2 (Ibanda and Nakasongola) where HPV vaccination of girls took place in 2008 and 2009 and 2 (Mbarara and Luwero) where it did not. Parents from districts where HPV vaccination of girls never took place were included for comparison purposes.

### Study population

Participants were recruited during the process of recruiting a corresponding sample of 10–23 year old 1,600 in-school boys into a related study. Data analysis for a separate manuscript from the 1,600 boys is on-going. A consent letter requesting for participation of parents and their sons into the two related studies was delivered to 1,600 parents. Parents of secondary school boys that consented were recruited. A parent was taken to be either biological or a guardian who was living with and taking care of the boy(s). In selecting parents, authors worked with secondary schools which had students that were trekking to and from home every school-day. Secondary schools in study districts fulfilling this description were listed and probability random sampling was applied to select those to participate. Parents were accessed through their sons in the age range of interest.

### Study Sample

Since variability in the proportion of parents willing to allow son(s) to receive HPV vaccines if availed in future was unknown, we adopted the most conservative proportion of 50% which represents maximum variability in any population. We set the desired confidence level at 95% and the level of precision at ±5%. Using the online *Raosoft* sample size calculator [31], we derived an estimated sample of 377 for each participating district, giving a total of 1,508 parents. Care was taken to have a sample of parents with a male to female ratio of about one. Parents that responded to the questionnaire were 870.

### The Research Tool

Basing on study objectives and literature review, a pre-coded self-administered questionnaire was developed and used to collect data from parents on their socio-demographic characteristics, knowledge about HPV and HPV vaccine, perception about their son(s)' risk of HPV infection, and willingness to allow probable HPV vaccination of boys in future. Socio-demographic data included: gender, domicile, educational attainment, religious affiliation, and occupation.

Knowledge was assessed by asking parents to choose one response from: 1 =  Yes, 2 =  No and 3 =  I don't know (if they were not sure) to each of these statements: *‘HPV is usually sexually transmitted’*, *‘Males cannot get HPV’*, *‘Condom use can prevent HPV’*, *‘Sexual abstinence prevents HPV’*, *‘HPV Vaccine effectively protects against HPV’*, *‘HPV Vaccine can cure HPV’*, *‘HPV Vaccine can cause infertility’*, *‘HPV vaccine protects against HIV’*, *‘HPV vaccine protects against STDs’*, *‘HPV infected young girls can develop cervical cancer’* and *‘A healthy looking person can have HPV’*. Parents were also asked about what they perceived to cause cervical cancer and genital warts. At analysis, the response of ‘I don’t know’ and ‘No’ were merged to form ‘No’.

Perceived son(s)' risk of infection with HPV was assessed by a set of 5 statements requiring responses on a 5-point Likert scale of: 1 =  strongly disagree, 2 =  disagree, 3 =  neither agree nor disagree, 4 =  agree and 5 =  strongly agree. The statements were framed in such a way that parental response referred to a son(s). Sample statements included: *‘I feel that chances are high that my son(s) can get the HPV’, ‘I am afraid that my son(s) might contract the HPV’, ‘I believe that my son(s) can be exposed to HPV infection if he engages in sexual intercourse’, ‘I believe my son(s) can get HPV even if he/they are only having sex with one partner’ and ‘My son(s) would rather have HIV than HPV’*. Parents were also asked about what they perceived to be the current likelihood of their son(s) to acquire HPV and other STDS.

Parents’ willingness to have their sons to be vaccinated against HPV was assessed using a set of statements that required responding to a 5-point Likert scale rating of: 1 =  not at all likely, 2 =  not likely, 3 =  somewhat likely, 4 =  very likely, 5 =  extremely likely. The statements were stated as follow; *‘In the future, how likely are you to . . . ‘to ask a health worker to give your son(s) the HPV vaccine?’, ‘do additional research to understand the HPV vaccine?’*, *‘discuss the usefulness of the HPV vaccine to your son(s) with health workers?’, ‘make an appointment to have your son(s) get the HPV Vaccine?’ and, ‘do nothing to have your son(s) get the HPV vaccine?’*. Parents were also asked whether they would recommend the HPV vaccine to sons of their friends or relatives, whether they would allow their own sons to be given the HPV vaccine, and who should receive the HPV vaccine. They were further asked to give reasons for their views. The dependent variable in the study was parental willingness to allow son(s) to be given current HPV vaccines in future.

### Data Collection

Each participating parent gave oral and written consent. School boys delivered the questionnaires and consent forms to parents and returned them filled to research assistants. Data from a few parents who were semi-literate or illiterate was collected with the guidance of a research assistant who oversaw filling of the questionnaire. Each question item in the questionnaire had either a *Luganda* translation (for central Uganda districts) or a *Runyankore* translation (for western Uganda districts) to help parents who did not understand English. *Luganda* and *Runyankore* are commonly spoken Bantu language dialects in the study areas. To attain face validity, feasibility visits to assess the appropriateness of the proposed study sites were done. This was followed by a pre-test of the research tool on a sample of articulate respondents (not part of this study), that had similar demographic characteristics as the targeted study participants. The pre-test phase also enabled assessment of the adequacy of the research tool, likely data analysis techniques and identification of appropriate strategies to recruit study participants. Appropriate changes were made in the research tool after the pre-test.

### Data capture processes

Data was collected in November, 2012. Four research teams were simultaneously deployed, one per district. The research assistants were professional health workers with prior experience in HPV-related data collection. They were given more training about the study, field surveys, dynamics of fieldwork, content of research instruments, and their ethical obligations as data collectors. They were asked to inform participating parents not to ask or allow anyone to help them respond to questions. Parents were requested to put a tick or circle on the response option that represented their thoughts. Parents were also promised and accorded confidentiality by delinking any identifiers from the data.

### Data Management and Statistical Analyses

Data in questionnaires were checked for completeness and consistency and entered into a computer using EpiData software. Double data entry was done by two independent data entry clerks and later, compared to clean out inconsistencies. Data was then exported to the Statistical Package for the Social Scientists (SPSS) version 11 for analysis. Frequencies and percentages of parents' socio-demographics, knowledge, risk perception and willingness to accept probable vaccination of young males with Human Papillomavirus (HPV) vaccines in future were produced. At a descriptive level, parents' socio-demographics, knowledge about HPV and HPV vaccine, and perception of son(s)' risk for HPV infection were compared using a 2-way contingency table analysis (Pearson's chi-square statistic) on the basis of willingness to allow sons(s) to receive HPV vaccines in future. Differences in age through means and standard deviations using a Student's *t*-test were evaluated. Binary logistic regression (backward stepwise) to adjust for possible interaction and confounding of different independent categorical variables was used in evaluation of their association with the dependent variable. Parental willingness (dependent variable) was entered as: ‘willing to allow a son(s) to be given the HPV vaccine’  = 1 and ‘not willing to allow a son(s) to be given the HPV vaccine’  = 0. Level of significance was set at *p*≤0.05 and corresponding Odds Ratios (with 95% confidence intervals) were generated.

## Study Findings

### Demographic Description of Respondents

Parents from districts where HPV vaccination of girls had taken place were 456 (52.4%) with a mean age of 40.8 (SD = 15). Those from districts where HPV vaccination of girls had not happened were 414 (47.6%) with a mean age of 40.6 (SD = 12.7). Parents that were willing to allow a son(s) to receive the HPV vaccine in future were 681 (78.3%) with a mean age of 41 (SD = 14). Those not willing to allow a son(s) to receive the HPV vaccine were 85 (9.8%) with a mean age of 39.5 (SD = 12.3). Parents that never responded to the question on willingness to allow a son(s) to receive the HPV vaccine were 104 (12%). Parental age did not significantly differ between districts where HPV vaccination of girls had occurred and districts where vaccination had not occurred.

There was no significant statistical difference between parents willing and parents unwilling to allow their son(s) to receive HPV vaccines in future on all the demographic variables considered. However, parents that had ever heard about cervical cancer, parents that had ever heard about a vaccine to prevent cervical cancer or HPV, those who thought that HPV infection was the major cause of genital warts, those who would allow their daughter(s) to be given the HPV vaccine, and those who would recommend the HPV vaccine to sons of friends/family members were significantly more likely to be willing to allow their son(s) to receive the HPV vaccine in future (**see** **Table 1**). Belonging to districts where HPV vaccination of girls had taken place did not translate into more parental willingness to allow son(s) to be vaccinated against HPV in future. The exception was that more parents from districts where HPV vaccination of girls had happened agreed with the assertion that HPV infection is a major cause of genital warts (76% vs. 60.4%, Crude OR: 1.72, 95% CI: 1.19–2.47, χ2  = 8.99, *p* = 0.003).

**Table 1:** Parents' Characteristics by Willingness to Accept Boys' HPV Vaccination.

Parents' socio-demographic characteristics		Total for response category	Willingness to allow a son(s) to be given the HPV vaccine		Unadjusted OR (95% CI)
			Yes	No	
		n (%)	n (%)	n (%)	
Gender [Willing = 675; Unwilling = 82]					
	Male	490 (64.7)	431 (63.9)	59 (71.9)	Ref
	Female	267 (35.3)	244 (36.1)	23 (38.1)	0.69 (0.40–1.74)
Home location [Willing = 675; Unwilling = 83]					
	Urban	264 (34.8)	236 (35.0)	28 (37.7)	Ref
	Rural	494 (65.2)	439 (65.0)	55 (66.3)	1.06 (0.64–1.76)
Highest educational attainment [Willing = 671; Unwilling = 85]					
	No formal education	36 (4.8)	30 (4.5)	6 (7.1)	Ref
	Primary education	227 (30.0)	210 (31.3)	17 (20.0)	0.41 (0.14–1.25)
	Secondary education	352 (46.6)	307 (45.8)	45 (52.9)	0.73 (0.27–2.08)
	College or University education	124 (16.4)	107 (15.9)	17 (20.0)	0.79 (0.26–2.49)
	I do not know	17 (2.2)	17 (2.5)	0 (0.0)	0.00 (0.00–1.91)
Religious denomination [Willing = 676; Unwilling = 85]					
	Protestant	302 (39.7)	268 (39.6)	34 (40.0)	Ref
	Catholic	305 (40.1)	274 (40.5)	31 (36.5)	0.89 (0.52–1.54)
	Muslim	63 (8.3)	57 (8.4)	6 (7.1)	0.83 (0.29–2.19)
	Adventist	21 (2.8)	18 (2.7)	3 (3.5)	1.31 (0.29–5.08)
	Pentecostal	66 (8.7)	56 (8.6)	10 (11.8)	1.41 (0.61–1.20)
	Traditionalist	2 (0.3)	1 (0.1)	1 (1.2)	7.88 (0.21–296.42)
	Other	2 (0.3)	2 (0.3)	0 (0.0)	0.00 (0.00–33.42)
Occupation/Source of income [Willing = 542; Unwilling = 68]					
	Casual laborer	327 (53.6)	293 (54.1)	34 (50.0)	Ref
	Retail business (petty trade)	212 (34.8)	187 (34.5)	25 (36.8)	1.15 (0.64–2. 06)
	Unemployed	32 (5.2)	29 (5.4)	3 (4.4)	0.89 (0.21–3.29)
	Others	39 (6.4)	33 (6.1)	6 (8.8)	1.57 (0.54–4.28)
Ever heard about an illness called cervical cancer? [Willing = 617; Unwilling = 76]					
	Yes	575 (83.0)	519 (84.1)	56 (73.7)	Ref
	No	118 (17.0)	98 (15.9)	20 (26.3)	1.89 (1.05–3.39)*
Ever heard of a vaccine to prevent cervical cancer or HPV? [Willing = 643; Unwilling = 81]					
	Yes	441 (60.9)	408 (63.5)	33 (40.7)	Ref
	No	283 (39.1)	235 (36.5)	48 (59.3)	2.53 (1.54–4.16)***
Would you allow your daughter to be given the HPV Vaccine? [Willing = 632; Unwilling = 79]					
	Yes	644 (90.6)	605 (95.7)	39 (49.4)	Ref
	No	67 (9.4)	27 (4.3)	40 (50.6)	22.98 (12.30–43.15)***
Do you think HPV infection is the major cause of genital warts? [Willing = 629; Unwilling = 74]					
	Yes	551 (78.4)	507 (80.6)	44 (59.5)	Ref
	No	152 (21.6)	122 (19.4)	30 (40.5)	2.83 (1.66–4.83)***
Would you recommend the HPV Vaccine to sons of your friends/family members? [Willing = 640; Unwilling = 82]					
	Yes	661 (91.6)	620 (96.9)	41 (50.0)	Ref
	No	61 (8.4)	20 (3.1)	41 (50.1)	31.0 (15.97–60.64)***
District of residence [Willing = 681; Unwilling = 85]					
	Where HPV vaccination of girls took place	398 (52.0)	357 (52.4)	41 (48.2)	Ref
	Where HPV vaccination of girls never took place	368 (48.0)	324 (47.6)	44 (51.8)	1.82 (0.74–1.90)

Significance set at: *p≤0.05; **p≤0.01, ***p≤0.001; Deficits in the sample for each group represents cases of non-response to different statements.

### Parents Views about Cervical Cancer and Probable HPV Vaccination of Boys

Parents were asked about the importance of the HPV vaccine for son(s), what they thought cervical cancer meant, how they thought HPV was transmitted, reasons they would consider to allow their sons to be given the HPV vaccine in future, reasons they would consider to recommend the HPV vaccine to sons of friends and family members, who they believed should be given the HPV vaccine, their perception of a possibility that their son(s) could get an HPV infection and what they thought were the chances that their son(s) could get an STD as displayed in Table 2. The aim was to understand their views concerning cervical cancer, probable HPV vaccination of sons and assessment of son's risk to HPV infection and infection with other STIs.

**Table 2:** Parents'/Guardians' Views Regarding Cervical Cancer and Probable Vaccination of Boys with HPV Vaccines.

Questions items		Total	Willingness to allow a son(s) to be given the HPV vaccine		Unadjusted OR (95% CI)
			Yes	No	
		n (%)	n (%)	n (%)	
#How important is the HPV Vaccine for son(s)?					
	Not at all important	26 (4.8)	19 (3.9)	7 (12.7)	Ref
	Not very important	19 (3.5)	14 (2.9)	5 (9.1)	0.97 (0.21–4.46)
	Somewhat important	46 (8.5)	38 (7.9)	8 (14.5)	0.57 (0.16–2.09)
	Very important	448 (83.1)	413 (85.3)	35 (63.6)	0.23 (0.08–0.65)***
#What is cervical cancer?					
	A cancer in the cervix/lower part of womb	455 (51.9)	403 (51.5)	52 (55.9)	Ref
	An invasive tumor affecting the cervix	174 (19.9)	157 (20.1)	17 (18.3)	0.84 (0.45–1.55)
	It is mainly caused by HPV	223 (25.5)	203 (25.9)	20 (21.5)	0.76 (0.42–1.35)
	It is caused by Hepatitis Viruses B and C	24 (2.7)	20 (2.6)	4 (4.3)	1.55 (0.43–5.06)
How is HPV transmitted?					
	Sexual intercourse	661 (93.1)	590 (93.7)	71 (88.8)	Ref
	Mother to child transmission	49 (6.9)	40 (6.3)	9 (11.3)	1.87 (0.81–4.21)
#Reasons parents would consider to allow their sons to be given the HPV vaccine					
	HPV vaccine has no side effects	303 (32.5)	295 (32.9)	8 (22.2)	Ref
	HPV vaccine is at a low cost	108 (11.6)	103 (11.5)	5 (13.9)	1.79 (0.49–6.20)
	Certainty of HPV Vaccine's effectiveness	304 (32.6)	298 (33.3)	6 (16.7)	0.74 (0.23–2.39)
	Time is available to take sons to get HPV Vaccine	39 (4.2)	32 (3.6)	7 (19.4)	8.07 (2.44–26.63)***
	To discourage early sexual debut and unsafe sex	178 (19.1)	168 (18.8)	10 (27.8)	2.19 (0.79–6.23)
#Reasons parents would consider to recommend the HPV vaccine to sons of friends and relatives					
	Yes, for safety reasons	205 (19.5)	195 (19.7)	10 (16.4)	Ref
	It can prevent HPV infection	330 (31.4)	312 (31.5)	18 (29.5)	1.13 (0.48–2.68)
	Because it can protect against cervical cancer	211 (20.1)	201 (20.3)	10 (16.4)	0.97 (0.36–2.59)
	May be but I am not sure	39 (3.7)	27 (2.7)	12 (19.7)	8.67 (3.12–24.31)***
	Yes, to prevent cancer	132 (12.6)	126 (12.7)	6 (9.8)	0.93 (0.29–2.86)
	Yes, to prevent cervical cancer	133 (12.7)	128 (12.9)	5 (8.2)	0.76 (0.22–2.49)
#Who should receive/be given the HPV Vaccine?					
	All females before their sexual debut	257 (22.7)	225 (21.8)	32 (31.1)	Ref
	All females	276 (24.4)	252 (24.5)	24 (23.3)	0.67 (0.37–1.21)
	All males	258 (22.8)	240 (23.3)	18 (17.5)	0.53 (0.27–1.00)
	All males before their sexual debut	226 (19.9)	215 (20.9)	11 (10.7)	0.36 (0.17–0.77)**
	People with multiple sexual partners	116 (10.2)	98 (9.5)	18 (17.5)	1.29 (0.66–2.51)
#What do you think are the current chances of your son(s) getting an HPV infection?					
	High	171 (25.4)	156 (26.0)	15 (20.3)	Ref
	Medium	151 (22.4)	143 (23.9)	8 (10.8)	0.58 (0.22–1.51)
	Low	182 (27.0)	159 (26.5)	23 (31.1)	1.50 (0.72–3.16)
	None	61 (9.1)	50 (8.3)	11 (14.9)	1.29 (0.91–5.71)
	Uncertain	108 (16.0)	91 (15.2)	17 (23.0)	1.94 (0.87–4.34)
#What do you think are the current chances of your son(s) getting an STD					
	High	208 (29.9)	190 (30.8)	18 (22.8)	Ref
	Medium	136 (19.5)	123 (19.9)	13 (16.5)	1.12 (0.49–2.49)
	Low	163 (23.4)	143 (23.2)	20 (25.3)	1.47 (0.72–3.04)
	None	65 (9.3)	57 (9.2)	8 (10.1)	1.48 (0.56–3.85)
	Uncertain	124 (17.8)	104 (16.9)	20 (25.3)	2.03 (0.98–4.22)

Significance set at: *p≤0.05; **p≤0.01, ***p≤0.001; **#**Total scores for parents willing to allow sons to be given the HPV vaccine was greater than 681 and those not willing greater than 85 because multiple responses were allowed.

There were no significant differences between parents willing and those unwilling to allow their son(s) to receive HPV vaccines on most responses. However, parents who considered HPV vaccines to be very important for boys were more likely to show willingness to allow son(s) to receive HPV vaccination (85.3% vs. 63.6%, Crude OR: 4.35, 95% CI: 1.54–11.92, χ2  = 8.87, *p* = 0.003). Parents who believed that all males should receive HPV vaccines before sexual debut were more likely to show willingness to allow son(s) to receive HPV vaccination (20.9% vs. 10.7%, Crude OR: 2.78, 95% CI: 1.31–6.03, χ2 = 7.62, *p* = 0.006). Also, parents who were unsure about recommending the HPV vaccine to sons of friends and relatives were significantly less likely to be willing to allow their own son(s) to receive the HPV vaccine in future (2.7% vs. 19.7%, Crude OR: 8.67, 95% CI: 3.12–24.31, χ2 = 23.71, *p* = 0.000]. Incidentally, parents who reported that they had time to take their son(s) to get the HPV vaccine were significantly less likely to be willing to allow the same son(s) to receive the HPV vaccine in future (3.6% vs. 19.4%, Crude OR: 8.07, 95% CI: 2.44–26.63, χ2 = 15.83, *p* = 0.000].

### Parental Beliefs and Attitudes about Cervical Cancer, HPV, HPV Vaccine and HPV Vaccination of Boys

On the basis of parental attitudes and beliefs towards cervical cancer, HPV, HPV Vaccine and HPV Vaccination of boys, there were not many significant differences between parents willing and parents unwilling to allow their son(s) to receive HPV vaccines in future (Table 3). However, parents who agreed with the statement that chances were high that their son(s) could contract HPV were significantly more likely to show willingness to allow their son(s) to receive HPV vaccination in future (35% vs. 17.3%, Crude OR: 2.72, 95% CI: 1.24–5.95, χ2 = 6.63, *p* = 0.010). Parents who were afraid that their son(s) might contract HPV were significantly more likely to show willingness to allow their son(s) to receive HPV vaccination in future (39.9% vs. 24.1%, Crude OR: 0.37, 95% CI: 0.17–0.81, χ2 = 4.26, *p* = 0.039). Parents who believed that their sons could be exposed to HPV infection if they had sexual intercourse were significantly more likely to be willing to allow their son(s) to receive HPV vaccination in future (39.9% vs. 25.6%, Crude OR: 0.46, 95% CI: 0.22–0.97, χ2 = 4.26, *p* = 0.039). Parents who were neutral with the possibility that their son(s) could contract HPV even if they had sexual intercourse with one partner were significantly more likely to be willing to allow their son(s) to receive HPV vaccination in future (16.4% vs. 12.7%, Crude OR: 0.14, 95% CI: 0.17–0.97, χ2 = 4.12, *p* = 0.042). Also, parents who strongly agreed that their son(s) could contract HPV even if they had sexual intercourse with one partner were significantly more likely to be willing to allow their son(s) to receive HPV vaccination in future (12.5% vs. 8.9%, Crude OR: 0.37, 95% CI: 0.14–0.99, χ2 = 3.87, *p* = 0.049).

**Table 3:** Beliefs and Attitudes about Cervical Cancer, HPV, HPV Vaccine and HPV Vaccination of Boys.

Questions items		Total	Willingness to allow son(s) to be given the HPV vaccine		Unadjusted OR (95% CI)
			Yes	No	
		n = (%)	n = (%)	n = (%)	
I feel that chances are high that my son(s) can get the HPV	Strongly disagree	110 (15.5)	94 (14.9)	16 (19.8)	Ref
	Disagree	152 (21.4)	127 (20.2)	25 (30.9)	1.16 (0.57–2.42)
	Neither agree nor disagree	137 (19.3)	115 (18.3)	22 (27.2)	1.12 (0.53–2.39)
	Agree	234 (33.0)	220 (35.0)	14 (17.3)	0.37 (0.17–0.85)*
	Strongly agree	77 (10.8)	73 (11.6)	4 (4.9)	0.32 (0.09–1.09)
I am afraid that my son(s) might contract the HPV	Strongly disagree	87 (12.4)	72 (11.6)	15 (19.0)	Ref
	Disagree	167 (23.8)	142 (22.8)	25 (31.6)	0.85 (0.39–1.81)
	Neither agree nor disagree	97 (13.8)	85 (13.7)	12 (15.2)	0.68 (0.28–1.66)
	Agree	267 (38.1)	248 (39.9)	19 (24.1)	0.37 (0.17–0.81)**
	Strongly agree	83 (11.8)	75 (12.1)	8 (10.1)	0.51 (0.19–1.39)
I believe that my son(s) can be exposed to HPV infection if he/they has/have sex	Strongly disagree	114 (16.3)	97 (15.6)	17 (21.8)	Ref
	Disagree	148 (21.1)	131 (21.1)	17 (21.8)	0.74 (0.34–1.61)
	Neither agree nor disagree	94 (13.4)	76 (12.2)	18 (23.1)	1.35 (0.62–2.97)
	Agree	268 (38.3)	248 (39.9)	20 (25.6)	0.46 (0.22–0.97)**
	Strongly agree	76 (10.9)	70 (11.3)	6 (7.7)	0.49 (0.16–1.41)
I believe my son(s) can get HPV even if he is/they are only having sex with one partner	Strongly disagree	104 (15.5)	83 (14.0)	21 (26.6)	Ref
	Disagree	138 (20.5)	123 (20.7)	15 (19.0)	0.48 (0.22–1.05)
	Neither agree nor disagree	107 (15.9)	97 (16.4)	10 (12.7)	0.41 (0.17–0.97)*
	Agree	242 (36.0)	216 (36.4)	26 (32.9)	0.59 (0.31–1.12)
	Strongly agree	81 (12.1)	74 (12.5)	7 (8.9)	0.37 (0.14–0.99)*
My son(s) would rather have HIV than HPV	Strongly disagree	178 (26.9)	150 (25.5)	28 (38.4)	Ref
	Disagree	131 (19.8)	119 (20.2)	12 (16.4)	0.54 (0.25–1.16)
	Neither agree nor disagree	140 (21.2)	129 (21.9)	11 (15.1)	0.46 (0.21–1.003)
	Agree	142 (21.5)	126 (21.4)	16 (21.9)	0.68 (0.33–1.38)
	Strongly agree	70 (10.6)	64 (10.9)	6 (8.2)	0.50 (0.18–1.35)

Significance set at: *p≤0.05; **p≤0.01, ***p≤0.001; Deficits in the sample for each group represents cases of non-response to different statements.

### Parents' Beliefs about the Likelihood of Future Vaccination of Boys with the HPV Vaccines

Parents responded to statements about their likelihood to accept HPV vaccination of their boys with HPV vaccines in future. As shown in Table 4, there were few significant differences between parents willing and those unwilling to allow their son(s) to receive HPV vaccines in future. Parents who were very likely (38% vs. 17.5%, Crude OR: 0.24, 95% CI: 0.10–0.58, χ2 = 11.62, *p* = 0.001) and those who were extremely likely (23.9% vs. 12.5%, Crude OR: 0.27, 95% CI: 0.10–0.72, χ2 = 11.62, *p* = 0.001) to ask heath workers to give their son(s) the HPV vaccine in future exhibited more willingness to allow their son(s) to receive HPV vaccination in future.

**Table 4:** Parents/Guardians' Beliefs about the Likelihood of Future Vaccination of Boys with the HPV Vaccine.

Questions/Items		Total	Willingness to allow a son(s) to be given the HPV vaccine		Unadjusted OR (95% CI)
			Yes	No	
		n (%)	n (%)	n (%)	
In future, how likely are you to ask a health worker to give your son(s) the HPV vaccine?	No at all likely	66 (9.4)	53 (8.5)	13 (16.3)	Ref
	Not likely	83 (11.8)	64 (10.3)	19 (23.8)	1.21 (0.51–2.89)
	Somewhat likely	145 (20.6)	121 (19.4)	24 (30.0)	0.81 (0.36–1.83)
	Very Likely	251 (35.7)	237 (38.0)	14 (17.5)	0.24 (0.10–0.58)***
	Extremely likely	159 (22.6)	149 (23.9)	10 (12.5)	0.27 (0.10–0.72)**
In future, how likely are you to try and learn more about the HPV vaccine?	No at all likely	51 (7.4)	42 (6.9)	9 (11.8)	Ref
	Not likely	67 (9.7)	60 (9.8)	7 (9.2)	0.54 (0.17–1.76)
	Somewhat likely	144 (20.9)	123 (20.1)	21 (27.6)	0.79 (0.32–2.05)
	Very Likely	233 (33.9)	215 (35.1)	18 (23.7)	0.39 (0.15–1.02)
	Extremely likely	193 (28.1)	172 (28.1)	21 (27.6)	0.57 (0.23–1.46)
In future, how likely are you to discuss the usefulness of the HPV vaccine to your son(s) with health workers?	No at all likely	61 (8.8)	52 (8.5)	9 (11.4)	Ref
	Not likely	65 (9.4)	49 (8.0)	16 (20.3)	1.89 (0.70–5.14)
	Somewhat likely	151 (21.8)	130 (21.2)	21 (26.6)	0.93 (0.38–2.37)
	Very Likely	232 (33.5)	214 (34.9)	18 (22.8)	0.49 (0.19–1.25)
	Extremely likely	184 (26.6)	169 (27.5)	15 (19.0)	0.51 (0.19–1.36)
In future, how likely are you to make an appointment to have your son(s) get the HPV Vaccine?	No at all likely	74 (10.7)	63 (10.2)	11 (14.3)	Ref
	Not likely	65 (9.4)	53 (8.6)	12 (15.6)	1.29 (0.49–3.47)
	Somewhat likely	147 (21.2)	123 (20.0)	24 (31.2)	1.12 (0.49–2.61)
	Very Likely	246 (35.5)	228 (37.0)	18 (23.4)	0.45 (0.19–1.08)
	Extremely likely	161 (23.2)	149 (24.2)	12 (15.6)	0.46 (0.18–1.19)
In future, how likely are you to ignore or disregard allowing or having your son(s) to get or be given the HPV vaccine?	No at all likely	172 (25.0)	151 (24.7)	21 (26.9)	Ref
	Not likely	144 (20.9)	126 (20.6)	18 (23.1)	1.03 (0.49–2.16)
	Somewhat likely	130 (18.9)	111 (18.2)	19 (24.4)	1.23 (0.60–2.52)
	Very Likely	156 (22.6)	145 (23.7)	11 (14.1)	0.55 (0.24–1.24)
	Extremely likely	87 (12.6)	78 (12.8)	9 (11.5)	0.83 (0.33–2.02)

Significance set at: *p≤0.05; **p≤0.01, ***p≤0.001; Deficits in the sample for each group represents cases of non-response to different statements.

### Knowledge about HPV and HPV Vaccination Issues according to Willingness to have Boys Given the HPV Vaccines

Parents responded to 12 knowledge statements about cervical cancer, HPV, HPV vaccine and probable HPV vaccination as presented in Table 5. There were few significant differences between willing and unwilling parents to allow their son(s) to receive HPV vaccines in future. Only parents who correctly knew that HPV is usually sexually transmitted (81.1% vs. 65.8%, Crude OR: 2.23, 95% CI: 1.31–3.80, χ2 = 9.17, *p* = 0.002), those who knew that males could acquire HPV (52.9% vs. 26.6%, Crude OR: 3.10, 95% CI: 1.79–5.42, χ2 = 18.37, *p* = 0.000), and those who knew that the HPV vaccine effectively protects against HPV (68.4% vs. 48.1%, Crude OR: 2.33, 95% CI: 1.42–3.81, χ2 = 12.19, *p* = 0.000) had significantly more willingness to allow their son(s) to receive HPV vaccines in future. Generally, considerable proportions of parents lacked factual knowledge on each of the statements used to assess knowledge in this study.

**Table 5:** Knowledge about HPV and HPV Vaccine by Parental Willingness to Allow Son(s) to be given Probable HPV Vaccine in future.

Knowledge statements		Total	Willingness to allow a son(s) to be given the HPV vaccine		Unadjusted OR (95%CI)
			Yes	No	
		n (%)	n (%)	n (%)	
HPV is usually sexually transmitted					
	Yes	572 (79.4)	520 (81.1)	52 (65.8)	
	No	148 (20.6)	121 (18.9)	27 (34.2)	2.23 (1.31–3.80)**
Males cannot get HPV					
	Yes	352 (50.1)	294 (47.1)	58 (73.4)	
	No	351 (49.9)	330 (52.9)	21 (26.6)	0.32 (0.19–0.56)***
HPV can be transmitted through skin-to-skin genital contact without sexual intercourse					
	Yes	192 (26.9)	173 (27.4)	19 (23.2)	
	No	521 (73.1)	458 (72.6)	63 (76.8)	1.25 (0.71–2.34)
Condom use can prevent HPV					
	Yes	323 (45.4)	288 (45.6)	35 (43.2)	
	No	389 (54.7)	343 (54.4)	46 (56.8)	1.10 (0.68–1.81)
Sexual abstinence prevents HPV infection					
	Yes	428 (59.9)	379 (60.0)	49 (59.8)	
	No	286 (40.0)	253 (40.0)	33 (40.2)	1.01 (0.61–1.65)
HPV Vaccine effectively protects against HPV					
	Yes	471 (66.1)	432 (68.4)	39 (48.1)	
	No	242 (33.9)	200 (31.7)	42 (51.8)	2.33 (1.42–3.81)***
HPV Vaccine can cure HPV in already infected individuals					
	Yes	206 (29.3)	189 (30.3)	17 (21.0)	
	No	495 (70.6)	435 (69.7)	60 (79.0)	1.53 (0.85–2.81)
HPV Vaccine can cause infertility					
	Yes	164 (23.4)	144 (23.3)	20 (24.4)	
	No	537 (76.6)	475 (76.7)	62 (75.6)	0.94 (0.53–1.67)
HPV vaccine protects against HIV					
	Yes	112 (15.7)	103 (16.2)	9 (11.1)	
	No	603 (84.4)	531 (83.8)	72 (88.9)	1.62 (0.75–3.58)
HPV vaccine protects against STDs					
	Yes	157 (22.3)	142 (22.8)	15 (18.8)	
	No	547 (77.7)	482 (77.2)	65 (81.3)	1.28 (0.68–2.41)
HPV infected girls can develop cervical cancer					
	Yes	396 (55.9)	360 (57.1)	36 (45.6)	
	No	313 (44.1)	270 (42.9)	43 (54.4)	1.59 (0.97–2.62)
A healthy looking person can have HPV					
	Yes	436 (62.5)	394 (63.5)	42 (53.8)	
	No	262 (37.5)	226 (36.5)	36 (46.1)	1.49 (0.91–2.44)

Significance set at: *p≤0.05; **p≤0.01, ***p≤0.001; Deficits in the sample for each group represents cases of non-response to different statements.

In a multivariate analysis, we assessed the association between different covariates and willingness to allow sons to receive the HPV vaccines in future. All the significant factors (p≤0.05 at bivariate analysis) associated with willingness to allow son(s) to receive the HPV vaccines in future were entered into a binary logistic regression. The final step in the model showed few significant differences between willing and unwilling parents. Acceptance of HPV vaccination of daughters (Adjusted OR = 9.97; 95% CI: 4.57–21.76, p = 0.000) being likely to recommend the HPV vaccine to son(s) of friends and relatives (Adjusted OR = 18.25; 95% CI: 8.32–40.04, p = 0.000) predicted parental willingness to allow son(s) to be given the HPV vaccine in future. Conversely, being unsure about whether to recommend the HPV vaccine to sons of friends and relatives (Adjusted OR = 0.94; 95% CI: 0.01–0.67, p = 0.02) and disagreeing with the possibility that their sons could be exposed to HPV infection if they had sexual intercourse (Adjusted OR = 0.24; 95% CI: 0.07–0.79, p = 0.02) predicted parental unwillingness to allow son(s) to be given the HPV vaccine in future.

## Discussion

Similar to what has happened in many countries, the introduction of HPV vaccination in Uganda targeted only girls. Concerted efforts were put in the preparation of the population before the HPV vaccination of girls [21], [22], [33]. This sensitization raised general awareness about HPV and its consequences in Uganda but left unanswered the question of why boys were not vaccinated against it, yet it was explained to be a sexually transmitted infection. Ideally, effective eradication of cancers associated with HPV should follow a strategy of vaccinating boys and girls [5], [6]. It was on the basis of the likely benefits associated with the strategy of HPV vaccination of boys and girls that this exploratory study was done. Probable parental willingness to allow son(s) to receive current HPV vaccines if they were availed in future was assessed.

High proportions of parents were willing to allow sons (78.3%) and daughters (90.6%) to receive HPV vaccines if availed. This is consistent with studies that have established high HPV vaccine uptake among comparable adolescent populations [12], [21], [22], [34]. The relatively high parental acceptability of hypothetically vaccinating their sons with HPV vaccines is also consistent with commentaries from elsewhere [10], [19], [37]. It has been argued that vaccinating boys could be a viable complement to HPV vaccination of girls to avoid stigmatizing females as a source of STIs, improve social acceptability of HPV vaccines, eradicate HPV, protect boys from HPV infection, reduce HPV transmission, increase herd immunity, and prevent other HPV associated diseases [19], [37].

The higher proportion of parents willing to allow daughters to receive current HPV vaccines compared to those willing to allow sons in this study may reflect differences in knowledge and risk-perception of HPV infections for males and females. The lower proportion of parental willingness to allow sons to receive HPV vaccines could have been related to the focus of information about HPV infection and HPV-associated diseases on young women and parents of girls during sensitization that was a precursor to the introduction of HPV vaccination in Uganda [33]. Similar to what has been observed elsewhere, this could have left most parents ill-informed about HPV infection and its consequences on males' health [32].

Parents that had ever heard about cervical cancer were 83% while those that had ever heard about the HPV vaccine were 60.9%. Those who believed that HPV infection was a major cause of genital warts were 78.4%. The proportion of parents that would recommend HPV vaccines to sons of their friends and relatives was as high 91.6%. More parents from districts where HPV vaccination of girls had happened accepted the assertion that HPV infection is a major cause of genital warts, possibly indicating the role of sensitization before introduction of the vaccine [12], [21], [22]. This is consistent with past studies that show parental favorability and endorsement of HPV vaccination of boys [10], [11], [25], [27]. Future success in attempts to vaccinate males against HPV in Uganda and other comparable societies should be anchored on sustained sensitization about the linkage between HPV infection and men's health in general and sexual health in particular [1], [7], [8], [24], [25], [35], [37].

Parents who thought that vaccination of boys against HPV was very important were more likely to show willingness to allow son(s) to receive HPV vaccines in future. This suggests that parental acceptability of HPV vaccination for boys would be high if targeted sensitization focused on benefits their sons would receive if they were to receive HPV vaccines in future and the protection this would give to their spouses [10]. Expectedly, more parents that believed they would not have time to take sons to get HPV Vaccines and those not sure if they would recommend the HPV vaccine to sons of friends and relatives showed unwillingness to allow their sons to get HPV vaccines in future. Similarly, more parents who believed that all males should get the HPV vaccine before sexual debut were unwilling to allow their sons to get HPV vaccines in future. These findings reinforce the position that HPV vaccination of males potentially attracts controversy in all societies [6], [13], [15], [19], [32], [37]. This justifies the need for effective information, communication and education if males in a developing country like Uganda were to be given current HPV vaccines.

Parents who agreed that chances were high for their son(s) to contract HPV, those who were afraid their son(s) might contract the HPV and those who were either neutral or strongly agreed with the possibility that their son(s) could contract HPV even if they had sexual intercourse with one partner were more likely to show willingness to allow their son(s) to receive HPV vaccines in future. Parental acceptance of HPV vaccination of boys against HPV seemed to be associated with son(s) risk to HPV infection.

Up to 36% of parents thought that their sons were at risk of contracting HPV. These parents either agreed that their sons had high chances of acquiring HPV or were apprehensive of the likelihood that their son(s) could contract HPV if they had sexual intercourse. Up to 27% agreed with the assertion that their son(s) would rather have HPV than HIV which is indicative of a low perceived severity associated with HPV infection. Being an STI, HPV could be associated with stigma and some parents could have perceived that vaccinating their son(s) against an STI would tantamount to condoning high risk sexual behavior, similar to what has been noted elsewhere [8], [37]. Parental acceptance of HPV vaccination of daughters and parental likelihood to recommend the HPV vaccines to son(s) of friends and relatives were associated with parental willingness to allow sons to be given the HPV vaccine in future. This is consistent with the view that parental consent is required for any HPV vaccination [37].

If HPV vaccination of males was to be complement that of females in a socio-economic setting like Uganda, it will be necessary to emphasize the protective effects of HPV vaccines against genital warts other than mainstreaming prevention of cervical cancer only. The view of parents preferring their children to receive HPV vaccines which protects against both cervical cancer and genital warts has been articulated before [8]. Besides, there are several reasons why vaccinating boys against HPV can be beneficial notably; the vaccine being able to prevent many HPV-related conditions, including penile, anal, and head-and-neck neoplasias; genital warts; and recurrent respiratory papillomatosis [9], [10].

Parents who knew that HPV is usually sexually transmitted, that males can acquire HPV, and that HPV vaccines effectively protects against HPV were more likely to allow their son(s) to receive HPV vaccines in future. This underscores the positive influence that provision of accurate information about the sexual transmission of HPV, HPV risk for boys and girls once they get exposed through sexual intercourse and efficacy of the HPV vaccine has on parental acceptance of HPV vaccination of boys. However, considerable proportions of parents lacked factual knowledge in this study, which is indicative of a huge information gap. This study showed that there were concerns about the safety, likely effects and cost of the HPV vaccines for males. Therefore, widespread parental sensitization about risks and consequences of HPV infections and benefits of HPV vaccination not only to females but for males as well if HPV vaccines are to be well accepted is necessary [16], [23]. More research from resource constrained societies is still needed [37].

Inherent methodological weaknesses that characterize often cross-sectional studies were in this study as well and should be borne in mind when interpreting these findings. We could only infer association and not causality in this study. We admit that there are no definitive inferences about the direction of observed relationships between covariates and parental willingness to accept HPV vaccination of their son(s) in future. There could be threats to validity and reliability of findings since questions to investigate all the differences between willing and unwilling parents, in districts where HPV vaccination of girls had or had not happened may not have been included in the questionnaire we used. Parents' responses were hypothetical since HPV vaccines for males were yet to be approved in the country.

By allowing some parents with modest schooling to respond to questions on their own, complexities related to distortions in response interpretation might have occurred. We tried to solve this by providing each parent a cover letter and assigning research assistants to guide data collection for parents that felt uncomfortable to do it alone. Each question in the questionnaire was in English with a translation into either *‘a Luganda’* or *‘a Runyankore’* dialect. The process of translation into local dialects was carefully done to avoid alterations in meaning and conceptualization. An already described process that ensures conceptual equivalence, cultural sensitivity and validity was followed [4], [18]. For parental views not predefined in the questionnaire, research assistants took detailed notes and ensured data credibility. The principal author dedicatedly supervised fieldwork.

The strength of this study was the relatively big sample size which implies that findings could be generalized with relative comfort to the parent population. Data that was collected and the subsequent interpretations were reasonably valid and reliable and should be taken as a starting point for further research in this area. Future studies, perhaps interventional in nature should adopt a longitudinal design to the assessment of parental willingness to allow sons to receive availed HPV vaccines in order to identify potentially modifiable risk factors.

### Conclusions and Implications

Successfulness of HPV vaccination in developing countries like Uganda heavily rely on parental acceptability of the exercise. As a complement to HPV vaccination of females, HPV vaccination of males could be an important public health strategy since HPV vaccines are known to be efficacious in men as well. Men are not only an important vector in the transmission of the virus but they too can develop genital warts and anogenital cancers as a result of HPV infection.

Contrary to expectation, parents' knowledge of HPV and HPV vaccines in general was not a strong predictor of parental acceptance of vaccination of sons against HPV in future. If HPV vaccination of males is to be adopted as a complement to that of females, sustained sensitization about usefulness of HPV vaccines is necessary to enhance their acceptability not only for girls but for boys too. This sensitization should emphasize the high vulnerability of boys and girls to HPV infection if they are exposed. Future attempts to vaccinate males against HPV in Uganda and other comparable countries should be anchored on the linkage between HPV infection, men's health in general and sexual health in particular. Besides, parents willing to let sons get HPV vaccines in future agreed that this should happen before sons' sexual debut.

On comparing HIV and HPV, parents seemed not to perceive the likelihood of HPV infection of their sons to be too severe. This could be indicative of lack of awareness about consequences of HPV infection. It is vital to raise HPV risk and severity perception among parents if acceptability of HPV vaccination for males is to complement that of girls. This is because parents who believed that their sons could be exposed to HPV infection if they had sexual intercourse and those who were apprehensive about the likelihood that their son(s) could contract HPV showed more willingness to allow sons to receive HPV vaccination in future.

Lastly, it is necessary to elevate parent's confidence in health workers if they are to accept current HPV vaccines for their male children. Apparently, parents who were likely to ask heath workers to give their son(s) HPV vaccines in future showed more acceptability. Secondly, parents that generally oppose vaccination seemed to be the ones likely to refuse letting their sons to receive HPV vaccines in future. Most parents that accepted HPV vaccination of daughters were also likely to recommend HPV vaccines to son(s) of friends and relatives as well as to allow their own son(s) to receive the HPV vaccine in future.
